# Post-sample aperture for low background diffraction experiments at X-ray free-electron lasers

**DOI:** 10.1107/S1600577517011961

**Published:** 2017-10-16

**Authors:** Max O. Wiedorn, Salah Awel, Andrew J. Morgan, Miriam Barthelmess, Richard Bean, Kenneth R. Beyerlein, Leonard M. G. Chavas, Niko Eckerskorn, Holger Fleckenstein, Michael Heymann, Daniel A. Horke, Juraj Knoška, Valerio Mariani, Dominik Oberthür, Nils Roth, Oleksandr Yefanov, Anton Barty, Saša Bajt, Jochen Küpper, Andrei V. Rode, Richard A. Kirian, Henry N. Chapman

**Affiliations:** a Center for Free-Electron Laser Scienece, Deutsches Elektronen-Synchrotron DESY, Notkestraße 85, 22607 Hamburg, Germany; b Department of Physics, Universität Hamburg, Luruper Chaussee 149, 22761 Hamburg, Germany; c The Hamburg Center for Ultrafast Imaging, Universität Hamburg, Luruper Chaussee 149, 22761 Hamburg, Germany; d European XFEL GmbH, Albert-Einstein-Ring 19, D-22671 Hamburg, Germany; e Laser Physics Centre, Research School of Physics and Engineering, Australian National University, ACT 2601, Canberra, Australia; f Photon Science, DESY, Notkestraße 85, 22607 Hamburg, Germany; g Arizona State University, Tempe, Arizona, USA

**Keywords:** X-ray diffraction, single-particle imaging, coherent diffractive imaging, aperture, background scattering, signal-to-noise ratio

## Abstract

The success of diffraction experiments from weakly scattering samples strongly depends on achieving an optimal signal-to-noise ratio. This is particularly important in single-particle imaging experiments where diffraction signals are typically very weak and the experiments are often accompanied by significant background scattering. A simple way to tremendously reduce background scattering by placing an aperture downstream of the sample has been developed and its application in a single-particle X-ray imaging experiment at FLASH is demonstrated. Using the concept of a post-sample aperture it was possible to reduce the background scattering levels by two orders of magnitude.

## Abstract 2

Diffraction experiments with weakly scattering samples often suffer from a low signal-to-noise ratio due to unwanted background scatter. Improving the signal-to-noise ratio for single-particle imaging experiments is particularly important as the diffraction signal is very weak. Here, a simple way to minimize the background scattering by placing an aperture downstream of the sample is demonstrated.

Achieving an optimal signal-to-noise-ratio is a key parameter in diffraction experiments with intense X-ray free-electron laser (FEL) sources. This can ideally be performed by reducing the level of background signal caused by stray light from upstream optics and slits. For diffraction experiments with low signal levels, beamlines must be optimized to reduce and stabilize background scatter in order to accurately measure small signal variations (Yun *et al.*, 1987 ▸; Miao *et al.*, 2003 ▸; Li *et al.*, 2008 ▸; Dufresne *et al.*, 2009 ▸; Kirby *et al.*, 2013 ▸). This is particularly relevant for X-ray imaging of isolated biomolecules and viruses (Seibert *et al.*, 2011 ▸), where the signal can be as little as a few hundred scattered photons per exposure (Yoon *et al.*, 2016 ▸; Ayyer *et al.*, 2015 ▸), or controlled-gas-phase-molecule diffraction, where signals can be <1 scattered photon per exposure (Küpper *et al.*, 2014 ▸).

A very intense X-ray beam is necessary to produce detectable patterns, which is accompanied by an increase of the scattering background and, therefore, does not necessarily improve the signal-to-noise ratio. As conventional notions of signal averaging and background subtraction are not effective in single-particle measurements, the reduction of the background scattering level appears essential. A common approach to reduce background scattering is the use of slits and apertures located upstream of the sample. Unfortunately, slits at the same time can act as secondary scattering sources when straddling the beam, and the optimization of slit size and position is often compounded by drifts and shot-to-shot jitter in the X-ray beam position and shape.

We present a simple way to significantly reduce the background in X-ray scattering experiments by using an aperture located downstream shortly behind the sample. Conceptually, this post-sample aperture (PSA) forms an opaque wall equipped with a pinhole letting the diffraction signal pass to the detector whilst blocking scattered X-rays from upstream apertures and focusing optics (see Fig. 1 ▸). The key to its effectiveness is the small angular size of the aperture with respect to the upstream scattering sources. Yet the aperture is much larger than the intense focal point of the X-ray beam and hence does not act as a secondary scattering source.

We first applied the PSA concept in single-particle imaging experiments at the FLASH FEL facility with 13.5 nm X-rays using a PI-MTE detector (Princeton Instruments). Fig. 2 ▸ shows uncorrected flat-field images for data collected with and without the PSA. The left-hand panel shows a single exposure without PSA integrated over ten pulses of 55.3 µJ average energy (1.98 × 10^13^ incident photons). The right-hand panel shows the background levels with PSA corresponding to 100 pulses of each 47.1 µJ average energy (1.69 × 10^14^ incident photons). Fig. 3 ▸ shows the radial distribution of the background derived from averaging over several patterns under comparable conditions, and normalized to the incident number of X-ray photons.

This dramatic reduction of the background scattering level is at least in part caused by the relatively large amount of upstream scatter present in this particular experiment, which was the motivation for introducing the PSA. Due to the effectiveness of the PSA, it has been replicated in more recent diffraction experiments using 70 nm viruses and hard X-ray FEL pulses (Munke *et al.*, 2016 ▸).

## Figures and Tables

**Figure 1:**
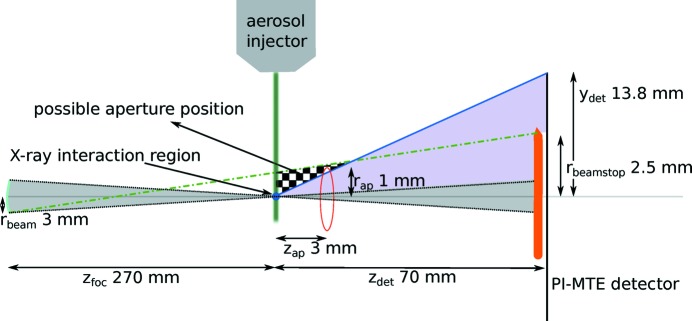
Schematic of the experimental setup showing relevant distances. The upstream scattering sources are modelled as an incoherent source of radius 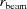 located a distance 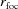 from the sample. This is the size and position of the beam at the last focusing optic, which acts as a source of scattered radiation of greatest angular extent when viewed from the sample. Given a beamstop of radius 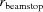 and a detector half-width 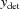, both located 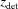 downstream of the sample, the checkered area shows the region where the aperture can be placed in order that the parasitic scattering does not reach the detector surface. The square aperture used here had a half-width of 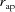 and was placed a distance 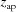 downstream of the sample position. Within the checkered region, the penumbra of the aperture from the incoherent source (indicated by the green dash–dot line) maps to the beamstop and the diffraction from the sample on the detector is not shadowed by the aperture (blue line and purple shaded area). The focused (coherent) X-ray beam is depicted by the grey shaded area. The aerosol injector, shown schematically, directs samples towards the beam focus. The diagram is not drawn to scale.

**Figure 2:**
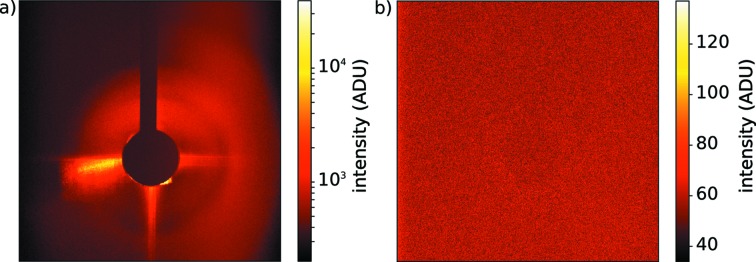
(*a*) Single detector readout before installing the PSA (logarithmic colour scale). The data collection was limited to ten pulses due to detector saturation. (*b*) Single detector readout after installing the PSA (linear colour scale). The background is flat and low in counts, especially in the central area around the beam stop. The full bunch train of 100 pulses could be used for this study.

**Figure 3:**
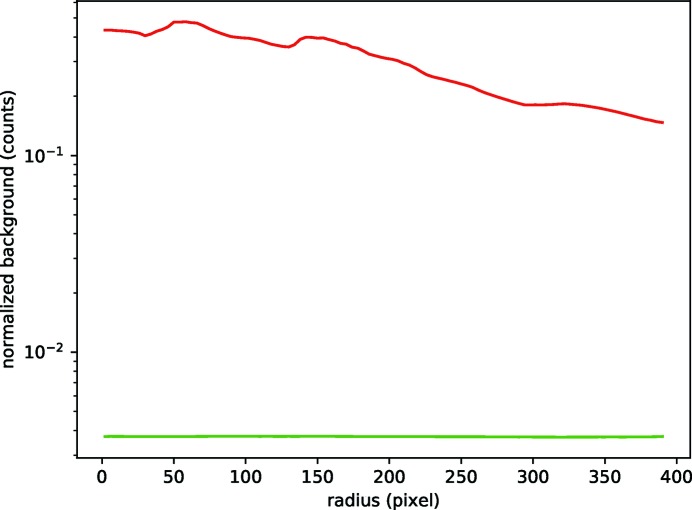
Radial average of 15 patterns before (red) and after (green) installing the PSA. The average background intensity normalized to the number of X-ray pulses and their respective intensities is two orders of magnitude lower with the PSA in place.
